# “Just Ask Me”: The Importance of Respectful Relationships Within Schools

**DOI:** 10.3389/fpsyg.2021.678264

**Published:** 2021-06-15

**Authors:** Charlotte Brownlow, Wenn Lawson, Yosheen Pillay, Joanne Mahony, Ding Abawi

**Affiliations:** ^1^School of Psychology and Counselling, University of Southern Queensland, Toowoomba, QLD, Australia; ^2^Centre for Health Research, Institute for Resilient Regions, University of Southern Queensland, Toowoomba, QLD, Australia; ^3^Curtin Autism Research Group, Curtin University, Perth, WA, Australia; ^4^School of Education, University of Southern Queensland, Toowoomba, QLD, Australia

**Keywords:** co-production, education, autistic identity, support, school, student experience, inclusion

## Abstract

An inclusive approach to education requires schools and educators to address the support needs and individual predispositions of all students. Our research highlights the crucial importance of effective and respectful communication with autistic students to facilitate their successful participation in schools. This paper explores the experiences of 24 autistic individuals aged 16–67 years, through synchronous semi-structured interviews and written responses. The research team comprised both autistic and allistic (non-autistic) researchers, who worked together to design the overall project, collect interview data, and analyse the data. Relationships were frequently discussed by participants and the importance of positive relationships was positioned as key to successful participation within educational contexts. Particularly damaging were assumptions made by teachers concerning individual ability based on labels given. Participants recalled ongoing challenges with resisting stereotypes and managing stigma, while trying to craft a positive autistic identity and advocate for rightful supports for their education. At the core of these negotiations were positive relationships, and teachers who asked participants what their needs were, and then listened and proactively responded to their answers. Recommendations for more positive schooling engagements with autistic young people are provided.

## Introduction

There is little debate in the literature that autistic people are neurologically different from allistic^1^ (non-autistic) people. However, autistic people have been positioned in research and clinical literature as “lacking,” with “deficits” in areas of communicative and social interactions, alongside restricted and repetitive behaviours and interests (American Psychiatric Association, 2013). While it is clear that autistic people do have difficulties with some aspects of their lives, these are *differences* not *deficits*. For example, autistic individuals tend to focus on specific areas of interest much more than allistic individuals, who generally connect to broader, less focussed input from their environment (Lawson, 2011: Mottron et al., 2006). When individuals diverge from the typical, according to traditional thinking, deficit language is often used, which can lead to increased ableism (see Botha et al., 2021; Bottema-Beutel et al., 2021). Over the past six decades, autism has been considered a “disadvantage” and a “disorder.” For example, it is common to read statistics such as “autism is a lifelong condition with estimated annual support costs to Australia potentially exceeding $7 billion” (Synergies Economic Consulting, 2011). Not seen however is any official costing or commentary on the personal, social, and/or economic impact on individuals, families, communities, and the nation of *not* adequately including or supporting autistic individuals in appropriate ways. For young people, schools are crucial environments in which to flourish both personally and academically. However, autistic young people are frequently met with exclusionary practices, pervasive discrimination, and bullying (Humphrey and Symes, 2010; Maïano et al., 2016).

Over the last decade, Australia has seen an increase in individuals recognised as being autistic, with 1 in 52 adolescents aged 13–15 years old identified, and an overall increase of 42% since 2012 (Autism Asperger's Advocacy Australia, 2015; Australian Bureau of Statistics, 2018). It is well-documented that autistic students^2^ experience difficulties; less well-known is what supports are required to improve this situation. Traditionally, the emphasis has been on changing the autistic student to fit the school system (Lilley, 2014). More recent research suggests that it is more helpful to consider autistic students as different and not deficient, and that communication is the responsibility of all involved (Crompton et al., 2020). Within Australian school systems, the push towards inclusive practices exists within formal legislation that differs across, and between, states With higher numbers of autistic individuals being identified, more attention needs to be provided to their supports in various contexts, including education.

An inclusive approach to education requires schools and educators to address individual preferences and support the needs of all students, including those who are autistic (Batten, 2005; Lynch and Irvine, 2009). According to the Australian Bureau of Statistics (2018), 40% of autistic students accessed special tuition, 32% had support from a counsellor or disability support person, and 28% did not receive any support (Australian Bureau of Statistics, 2018). These statistics suggest that currently, autistic students require additional support to engage with and be successful at school, because the existing educational system does not provide adequate support to nurture all young people (Wei et al., 2014). Recent research with educators, parents, specialists, and autistic students explored support to improve educational outcomes for autistic students, found a lack of funding, limited education and training, time, and specialist support as barriers to supporting needs of autistic students (Saggers et al., 2016). Interviews with teachers and parents identified that the least supported needs of autistic students in educational settings were social and emotional, followed by behavioural, communication, and sensory needs.

It is therefore crucial that research explores both the barriers and enablers to positive educational experiences. We argue that this research needs to be underpinned by two main concepts:

1. An understanding that interactions between autistic and allistic people can be challenging due to inherent differences in neurology, with neither one being better or worse, but both being important aspects of human neurodiversity (see Singer, 1999; Milton, 2012); and
2. Not presuming that autistic equals deficient and, therefore, inferior, or that allistic equals acceptable and, therefore, superior.

Informed by the above, the current research sought to understand the school experiences of autistic young people and adults, adopting an abilities framework, i.e., focusing on *differences* not *deficits* and working from a presumption of “competence” rather than “incompetence” for each participant. The research team comprised both autistic and allistic researchers, who worked together to design the overall project, collect interview data, analyse the data, and write up the findings.

## Method

### Research Design Overview

A central feature of the research design was flexibility to enable participants to contribute to the data in ways that were accessible for them. Participants were able to contribute to an individual semi-structured interview, provide a written account, or engage in a text-based individual interview. Nineteen participants chose a semi-structured interview which was conducted either via Zoom or in person with a member of the research team, five participants chose to contribute via a written account and no participants elected to provide a text-based interview. A clear interview protocol was developed, that included prompt suggestions for interviewers (see Appendix). We were particularly interested in educational experiences within schools so we were looking for participants to share their experiences of what they found helpful in supporting their education and also elements that proved to be challenging for them. As such, the interview focused around seven primary open ended questions. However, given the semi-structured design, interviewers responded to participant answers flexibly and asked additional follow up questions where considered appropriate in order to capture fuller understandings of participants' experiences.

### Recruitment

Following approval from the host university's Human Research Ethics Committee, an advertisement for the research was circulated via social media calling for participants. Interested individuals contacted a member of the team and were forwarded further information. This informed them of the study's requirements, participation options, participation incentives, and dissemination of study findings. Participants were then sent a consent form, with those 16–18 years sent an assent form and a consent form for parents to complete. On receipt of a signed consent form, a mutually agreeable time for interview, either face-to-face or via Zoom, was arranged. Participants electing to make a written submission sent these to a member of the research team within a time frame convenient to them. Following the interview, all participants were sent a $30 electronic gift card as acknowledgement of their contribution. All interview recordings were professionally transcribed verbatim, and participants created a pseudonym for use in reporting of findings.

### Participants

Twenty-four participants elected to contribute to the research. Participants were required to identify as autistic, be over 16 years of age, and be willing to share their experience of the Australian education system with the research team. Participants were not required to have a formal clinical diagnosis. This reflects the position of the research team in understanding autism as a core part of identity and also in recognition of the systemic barriers to seeking and acquiring a formal diagnosis, including availability of clinicians and financial constraints. The research team elected to interview a large participant sample so diverse experiences of educational contexts were captured (see Table 1).

**Table 1:** Summary of participants.

Participant^*^	**Age**	**Age of formal identification**	**Response type**	**Education state**	**Type of school**
Aliya	23	Primary school	Interview	VIC	State; Private
Annie	44	43	Interview	UK; VIC	State; Home school; Travelling teacher
Bellae	18	15	Written	QLD	Private
Bobby	17	Primary school	Interview	QLD	Catholic; State
Cassie	41	37	Written	NT; NSW	State; Catholic
Damian	Early 30s	Late teens/early 20s	Interview	QLD	State
Dave	27	3	Interview	VIC	Special school
Dianne	41	37	Interview	VIC; QLD	State
Ella	16	Primary school	Interview	QLD	State
Freya	19	3	Interview	VIC	State
Gayle	35	Self-identified in adulthood	Interview	VIC	State
Helen	19	10	Interview	VIC	State
HY	22	3	Written	VIC	Special school; State
Jack	25	11	Interview	VIC	Catholic; State
Jacob	28	3	Written	TAS	State
Jane	46	44	Interview	VIC	Progressive kindergarten; Private
Jimmy	32	7	Interview	VIC	Catholic
Johnny	16	Before primary school	Interview	QLD	State
Kate	19	12	Interview	NSW	State; Catholic
Kwamay	43	39	Interview	VIC	State
Max	31	18/19	Interview	VIC	State
Pete	67	Not provided	Written	VIC	State
Sam	25	21	Interview	TAS	State
Shannon	19	3	Interview	VIC	State

* *All names are pseudonyms*.

Participants ranged from 16 to 67 years and had attended school in Australia. Participants had experiences with a range of school environments including mainstream state school, mainstream Catholic school, travelling teacher education, and specialist schools.

### Approach to Data Analysis

The research team adopted a reflexive framework for thematic analysis as outlined by Braun and Clarke (2006, 2019), drawing on Willig's (2013) concept of “empathic” interpretations of the data. An open and reflexive approach to inductive analysis was therefore adopted rather than the development of an apriori coding framework. This allowed for the experiences of the individual participants to be considered in their entirety rather than prioritising particular narratives. Following verbatim transcription, each interview or written response was coded individually by the whole research team. Individual coding was discussed by all researchers and then broader themes were extrapolated. The broader themes were then discussed by the whole team, with agreement being reached concerning themes to be prioritised. The prioritised themes were then drafted by the first author, with finalisation of thematic selection discussed and amended by the entire team.

## Findings

Three themes from the findings are prioritised for discussion in this paper. These are *Avoiding assumptions of ability: The need for effective communication*; *The dangers of stereotypes, stigma, and judgements; Fostering skills of advocacy*. The key themes are shown in Table 2.

**Table 2:** Thematic summary and definition.

**Main theme**	**Thematic summary**	**Example quote**
Avoiding assumptions of ability: The need for effective communication	This theme reflected the ongoing challenge of effective and respectful communication between teachers and their autistic students. Through clear two-way communication misassumptions concerning abilities could be avoided.	“…So there were times when it's like if you just asked me, I could clarify things…”
The dangers of stereotypes, stigma, and judgements	This theme reflected the impact of misassumptions on autistic individuals and the negative impacts that stereotypes can have on individuals.	“…they treated me as someone with a label. They treated me as if I couldn't do anything…”
Fostering skills of advocacy	This theme reflected the importance of fostering advocacy skills and allowing an individual to claim a positive autistic identity.	“…I realised that being different was a really amazing thing to be because I was already different before I was labelled…”

Several participants reported quite different experiences across primary and high school contexts, with a common report of increased challenges at high school compounded by increased experiences of stigma and negative perceptions of autistic differences. Relationships were frequently discussed by participants and the importance of positive relationships was positioned as key to successful participation within educational contexts. Particularly damaging were assumptions made by teachers concerning individual ability based on participant labels. Participants recalled ongoing challenges with resisting stereotypes and managing stigma, while trying to craft a positive autistic identity and advocate for rightful supports for their education. At the core of these negotiations were positive relationships and teachers who asked participants about their needs and listened and proactively responded to their answers. The following themes are proposed to capture the key elements underpinning the participants' reported experiences of schooling. At the core of these themes are the relationships that are built between students and their teachers and peers.

### Theme 1: Avoiding Assumptions of Ability: The Need for Effective Communication

Several of the participants recalled instances where teachers had made assumptions about their abilities and needs without asking the students. Sometimes participants reported that teachers seemed to believe they were an autism expert based on their previous experience of teaching an autistic student. Aliya sums up the need for open communication when recounting her experiences at school:

*So the big one was asking me. What I found out was they only did what they heard or had written and they wouldn't ask me how that made me feel or what would help me…So there were times when it's like if you just asked me, I could clarify things…if the teachers and students had asked me questions of what they were unsure of, that would have helped me a lot because it meant that they were interested and they wanted to help…So not checking on me, not asking questions and then having a belief and false facts already implemented. Aliya, 23 years*.

The lack of communication frequently meant that decisions were made at higher levels, with students being informed about decisions rather than being included in discussions. This held the expectation that they would follow whatever had been decided. For example:

*So basically, what I felt was that they expected me to trust that they had my interest at heart, that they weren't trying to annoy me, but they were trying to be helpful. But they were also telling me that you can't say that or talk to us like that, because it makes—we feel as though you're getting angry at us, criticising us. Basically, they're expecting me to give them the benefit of the doubt, but I don't think they were giving that to me. I think if there's going to be trust…expectations have to be equal on both sides…It felt like I was expected to do all the work, express myself perfectly and also trust everyone and never think bad thoughts, ill of them, never mistrust them…I think students disengage when they feel like school is against them. Jack, 25 years*.

For students like Jack this meant that he felt communication was one-way, directed *at* him rather than *with* him, with expectations that he would unquestioningly follow what had been agreed without his input or consent.

When teachers did communicate with students, it was also considered crucial to consider the impact of the words used. Part of this was the need to engage in more discrete communications. For example:

*I think that there were times when I was singled out and things were said to me in front of other kids that probably could have been saved for a quiet moment or not been bothered to be dealt with at all. Kwamay 43 years*.

Similarly, the importance of carefully constructed written communications was also highlighted. For example:

*I look at my reports and a lot of reports talk about me being interrupting, me speaking out of turn, me talking when not spoken to and being an obstructive person. But they also talk about not trying hard enough, not putting in my best effort and giving up…maybe my mum shouldn't have let me read my reports and just sort of congratulated me that I was doing the best I could. But we did talk them through, and my mum would say, I know you're doing your best, and I know it's hard, and that was great validation and mum really helped with my self-esteem. But the damage is already done to some degree because that person thinks I'm not trying hard. Annie, 44 years*.

Instead of negative communications such as the examples above, participants argued that more positive and respectful communications that can promote positive relationships were needed. Jane provided the following example:

*I shot up the reading groups and my Grade 2 teacher said to me, look, I'd like to put you up into the top reading group but it's getting a bit full and I need the reading groups to have roughly even numbers. So, let me tell you that you are ready to move into the pink group but you're staying in the red group…I could accept that because she talked to me like a reasonable adult. She treated me like a sensible person, and it was a perfectly reasonable excuse. I knew there couldn't be 12 people in the pink group when there were 22 people in the class. Jane, 46 years*.

Jane's example demonstrates that clear communication that is positive and validating for the individual, while still managing the mechanics of a busy classroom, is possible if teachers believe in its importance, prioritise its use, and have the necessary skills. The second element that was central to positive relationships was the suspension of stereotypes and judgements that lead to stigma.

### Theme 2: The Dangers of Stereotypes, Stigma, and Judgements

Participants reported several instances of presumptions about their abilities by others in their reflections on school. For some, the assumptions that accompanied their diagnostic label/s overshadowed the reality of the individual's abilities and strengths. For example:

*For some time I wasn't even able to use proper scissors because they thought I would cut myself and stuff like that. That was mostly substitute teachers or teachers who didn't really understand. But yeah, the label kind of got in the way of some of that…They kind of knew I was different. They kind of knew I had a label. So in such, they treated me as someone with a label. They treated me as if I couldn't do anything. Aliya, 23 years*.

These assumptions often reflected what was perceived to be a rigid system, where understanding of difference and diversity was limited. Such assumptions sometimes involved an incorrect notion of an autistic person's nature, while at other times an inappropriate use of language and stereotyping of a child's abilities. For example:

*It's like, well, females, you can't be autistic. It was a male thing. If they ever heard of females on the spectrum it was always they're shy, they don't talk to people. They don't like being around other people. They won't speak to you. Most of them are non-verbal. They're not outgoing, they're not boisterous. They won't be able to process things…So it was kind of like the total opposite of what I was…I wasn't really treated like another person. I was treated very carefully. Aliya, 23 years*.

*There was a time in a class where I, for whatever reason, a frustration was building up over feeling like I was being patronised, by students in the class, teachers. I'm sure they wanted to be helpful, but what I received was they thought that I was incompetent I suppose, I need to be walked through everything and couldn't do things by myself. Jack, 25 years*

*I was at Year 12 camp. I was eating outside because it was too loud. This lady comes up to me, hellooo. Are you eating out—why are you talking to me so slow? I think the issue was…people don't seem to understand that autistic children become autistic adults. It's not like you grow out of it. Kate, 19 years*.

With assumptions comes stigma, and most participants reflected on feeling stigmatised at times during their schooling, sometimes leading to bullying.

*There was a definite kind of stigma, because it was like there was no complexity around the designation, it was just “special kid.” Damian, 30 years*.

Negative experiences were not confined to stereotyping and stigma; they were also an issue when advocating for supports within the school system. Several participants chose to act as if they were allistic, but found this an exhausting act to maintain, and one that many said should not be required of individuals also managing the social and academic challenges that all students encounter within schools.

*At first, there wasn't a lot of accommodation… that was mostly because of me; I didn't really want to talk about having a diagnosis…the counsellors in my primary school, kind of made me feel like it was something to be ashamed of. That I just couldn't tell people. It was like a horrible secret. Essentially, I just muscled through it and acted like I was neurotypical, for 3 or 4 years. Eventually, I just caved and couldn't do it anymore. Kate, 19 years*.

What is clear from these quotes is that the pretence of “normality” is the underpinning, yet unstated, concept on which school culture is based, with those unable to enact this explicitly singled out as different.

### Theme 3: Fostering Skills of Advocacy

Many of the participants, who were now adults, reflected on their time in school and the shifts that they have subsequently made towards positively claiming their autistic identity. Dave, however, reflects on the powerless position that students can find themselves in within educational contexts, which needs to be challenged in order to achieve advocacy:

*I mean pretty much everyone was powerless so, yeah, we couldn't—we really had to do everything as asked…Because actually I would have loved to have a lot more choice but unfortunately they really just wanted us to do this and that, and this and that, as the way they wanted us to do things. Dave, 27 years*.

For some, high school was the time that they began to positively take ownership of their label and positively embed this within their identity. For example:

*Well I think by the time I was in high school the Asperger's label was really, really something I'd embraced… something I liked talking about, it's something I was very comfortable identifying with. I think I was aware that some people on the spectrum didn't like a label, didn't like labels. But for me I think it was something I needed to make it concrete, to make it feel real. Jack, 25 years*.

The culture of the school and the position set by school leadership was, however, seen as central in facilitating this positive identity formation. Also, the school's willingness to promote and encourage self-advocacy programs had important impacts on individuals. For example:

*So we were encouraged to speak out if there was something that we were unsure of, and then they'd make changes…I was encouraged to be part of leadership boards, attend leadership training and things to foster self-advocacy skills…I used to be really uncomfortable reaching out when an issue arose. But now I feel a lot more comfortable and I know what to do if there's a situation that requires me to advocate. Freya, 19 years*.

*It wasn't until I got older when I realised that it was a lot of just self-talk. The fact is that it was okay to be different. I started to see myself a little differently. It wasn't until Year 12 when I was introduced to the ICAN program that I realised that being different was a really amazing thing to be because I was already different before I was labelled. Aliya, 23 years*.

Most participants, when reflecting on their experiences in school, spoke of being framed by others' assumptions of their abilities based on the label/s given to them. For some, the claiming of their labels as integral parts of their positive self-identity was delayed but, for others, it was actively facilitated by schools through introduction to peer mentoring programs such as those run by the ICAN network (see: https://icannetwork.online/). The role of peers in providing positive models for young people has attracted increased attention in recent years. Participants contributing to our research who had participated in such programs, repeatedly reflected on the positive outcomes they gained from this mentoring. However, while largely positive moves in self-identity were made during the latter years at school, the general experience of the school environment for some remained a challenge.

## Discussion

The three core themes presented in this paper reflected the experiences reported by the participants in their communicative interactions with teachers and peers and the damaging effects of assumptions, stigma, and stereotypes about both them as individuals and autistic people more broadly. While participants shared their stories of both positive and negative encounters within school and the effects that these had on them as individuals and their identities, those who had left school also provided reflections of the effects of these post formal schooling. While some participants reported very negative experiences at school, with their competence continually being questioned by peers, teachers, and the broader system, all reported their current situation as being more positive, reflecting the importance of the nurturing of advocacy skills and positive identity development for young people. Many who were told they were not academic enough to complete an academic high school pathway are now at university studying for a range of degrees. Some are researchers, some are teachers, and some have taken a more applied pathway via the Australian further education college system (TAFE). All participants were passionate about the need to support autistic young people still at school, and some had taken on mentorship roles to provide autistic role models for young people.

A core facilitator for enabling positive educational experiences was the establishment of positive relationships and respectful communication between teachers and students (Theme 1). Effective communication was considered a two-way process, with the need for teachers and others to suspend some of their previous assumptions concerning the capabilities of autistic people, and listen and respond to students as individuals according to the needs for support articulated by them. Key barriers to positive educational engagement that were reported by the participants included negative stereotypes and assumptions about the capabilities of individuals, and not creating a safe space for students to identify, communicate, and/or access appropriate supports, as reflected in Theme 2. Theme 3 outlined the need to create an environment within which self-advocacy skills could flourish. Such an environment is unlikely to be successful without a critical reflection on previously held attitudes and beliefs concerning the abilities of autistic people. For such changes in thinking to occur, positive relationships are likely to be key in effectively supporting autistic students within the classroom. Autistic students should not be pre-judged by their label and educators need to proactively ask, listen, and respond to autistic students and the experiences they have to share.

In summary, this research has highlighted some of the key challenges encountered by autistic students within schools. We hope that future research, using a strengths-based approach that acknowledges differences rather than deficits, will investigate further ways of improving the school experience for autistic students. Based on the three key themes identified in our research, we recommend the following points as positive ways forward within schools in creating supportive and inclusive classrooms:

- Prioritise relationships with autistic students;
- Proactively ask, listen, and respond to autistic students and the experiences they have to share**;**
- Create a safe environment that facilitates conversations, understanding of students' needs, and offers meaningful choices to students;
- Presume competence;
- Remember that every autistic person is different—don't make assumptions based on a label.

## Data Availability Statement

The raw data supporting the conclusions of this article will be made available by the authors, without undue reservation.

## Ethics Statement

This study involving human participants was reviewed and approved by the Human Research Ethics Committee, University of Southern Queensland. Written informed consent to participate in this study was provided by the participant/participants' legal guardian/next of kin.

## Author Contributions

CB, WL, and YP wrote the first draft of the manuscript and JM and DA refined and edited. All authors contributed to the design, data collection, and analysis for this paper.

## Conflict of Interest

The authors declare that the research was conducted in the absence of any commercial or financial relationships that could be construed as a potential conflict of interest.

## Footnotes

^1^The term allistic has been chosen to describe non-autistic people. Derived from Greek, the terms “allistic” and “autistic” are neutral and non-judgemental descriptors.

^2^The term “students” throughout the paper refers to individuals who are in school environments including primary, middle, and high school.

## Appendix

The key questions used in the interviews were:

1. Please tell us a little bit about yourself.
2. Please tell us about a really good teacher who has taught you.
3. In terms of your experience of education, were there things that could have been done differently?
4. What supports were put in place for you during school?
5. How did your experiences affect how you felt about yourself?
6. Please tell us about any non-academic challenges that you faced at school.
7. Is there anything else that you would like to add that you think is important that we know?
